# Supplementary material for: MetaRibo-Seq measures translation in microbiomes
Source: Nat Commun. 2020 Jun 29;11:3268. doi: 10.1038/s41467-020-17081-z (PMC7324362; doi:10.1038/s41467-020-17081-z)
Supplement: Supplementary file 10 — Supplementary Data 7 [file 41467_2020_17081_MOESM10_ESM.zip › File2/Confidence_High_Taxonomy/260667_out.krona.html]

Javascript must be enabled to view this page.

members
magnitude
magnitudeUnassigned
count
unassigned
taxon
rank

260667\_out

7

2
superkingdom
7

201174
7
phylum

1760
class
7

order
7
85004

31953
family
7

1678
genus
7

4
species
1680

SRS017103\_contig\_number\_42991SRS050752\_contig\_number\_contig-100\_947.165067SRS076756\_contig\_number\_21849SRS146812\_contig\_number\_44334

species
3
28026

SRS012902\_contig\_number\_1357SRS023829\_contig\_number\_contig-100\_178.178SRS075398\_contig\_number\_1604
